# Gut–Brain Axis and Neurodegeneration: State-of-the-Art of Meta-Omics Sciences for Microbiota Characterization

**DOI:** 10.3390/ijms21114045

**Published:** 2020-06-05

**Authors:** Bruno Tilocca, Luisa Pieroni, Alessio Soggiu, Domenico Britti, Luigi Bonizzi, Paola Roncada, Viviana Greco

**Affiliations:** 1 Department of Health Sciences, University “Magna Græcia” of Catanzaro, viale Europa, 88100 Catanzaro, Italy; tilocca@unicz.it (B.T.); britti@unicz.it (D.B.); 2 Proteomics and Metabonomics Unit, Fondazione Santa Lucia-IRCCS, via del Fosso di Fiorano, 64-00143 Rome, Italy; l.pieroni@hsantalucia.it; 3 Department of Biomedical, Surgical and Dental Sciences- One Health Unit, University of Milano, via Celoria 10, 20133 Milano, Italy; alessio.soggiu@unimi.it; 4 Department of Veterinary Medicine, University of Milano, Via dell’Università, 6- 26900 Lodi, Italy; luigi.bonizzi@unimi.it; 5 Department of Basic Biotechnological Sciences, Intensivological and Perioperative Clinics, Università Cattolica del Sacro Cuore, Largo F. Vito 1, 00168 Rome, Italy; 6 Fondazione Policlinico Universitario Agostino Gemelli, Largo A. Gemelli, 8-00168 Rome, Italy

**Keywords:** gut–brain axis, gut microbiota, meta-omics sciences, neurodegenerative diseases

## Abstract

Recent advances in the field of meta-omics sciences and related bioinformatics tools have allowed a comprehensive investigation of human-associated microbiota and its contribution to achieving and maintaining the homeostatic balance. Bioactive compounds from the microbial community harboring the human gut are involved in a finely tuned network of interconnections with the host, orchestrating a wide variety of physiological processes. These includes the bi-directional crosstalk between the central nervous system, the enteric nervous system, and the gastrointestinal tract (i.e., gut–brain axis). The increasing accumulation of evidence suggest a pivotal role of the composition and activity of the gut microbiota in neurodegeneration. In the present review we aim to provide an overview of the state-of-the-art of meta-omics sciences including metagenomics for the study of microbial genomes and taxa strains, metatranscriptomics for gene expression, metaproteomics and metabolomics to identify and/or quantify microbial proteins and metabolites, respectively. The potential and limitations of each discipline were highlighted, as well as the advantages of an integrated approach (multi-omics) to predict microbial functions and molecular mechanisms related to human diseases. Particular emphasis is given to the latest results obtained with these approaches in an attempt to elucidate the link between the gut microbiota and the most common neurodegenerative diseases, such as multiple sclerosis (MS), Alzheimer’s disease (AD), Parkinson’s disease (PD), and amyotrophic lateral sclerosis (ALS).

## 1. Introduction

In the last 15 years, the growing awareness of the sustained association between the intestinal microbiota and human health has led to many efforts to better understand its role and contribution in the pathogenesis of various diseases. 

At birth human intestine is essentially sterile; the onset of the gut microbiota starts as a dynamic ecosystem where the microbial composition increases both its diversity and richness until achievement of the “mature” level in the adult [1]. At this stage, the gut microbiota is a rather heterogeneous population including bacteria, fungi, archaea, viruses, and protozoa to an overall extent of about 10^14^ cells, approximately 10 times the number of cells of the human body [2]. Since its onset in the early life, the microbiota development and composition are influenced by several host-related variables (e.g., natural childbirth or caesarean section, genetic background, gender, age) and environmental parameters, such as dietary habits [1,3].

Bacteria is the most represented kingdom in the gut-associated microbial community and, although featured by a high inter-individual variability, a balanced composition of the human gastro-intestinal tract (GIT) microbiota is mainly represented by *Firmicutes* and *Bacteroidetes* phyla, and, to a lesser extent, by *Actinobacteria*, *Verrucomicrobia, Proteobacteria, Fusobacteria*, and *Cyanobacteria* phyla [4]. Bioactive compounds arising from the microbial commensals are involved in a finely tuned network of interconnections between the host and its microbiota and among microbiota members. Several lines of evidence have shown the ability of the microbiome to transmit signals molecules and metabolites of microbial origin to distant organs such as the brain [5,6,7], orchestrating a wide variety of physiological processes, ranging from normal homeostasis, host metabolism and immune system to brain functions. This close interconnection is also known as “gut–brain axis” (GBA).

In this view, accumulating data suggest that alteration in the optimal microbiome composition and activity (a condition named “microbiome dysbiosis”) may contribute to the onset of several pathologic conditions, such as neurological and neurodegenerative disorders [8,9]. 

The technological progress witnessed in the last two decades has marked a profound change in the methods employed for the study of the human microbiota and its pivotal role in both physiologic and pathologic processes. To date, studies based on germ-free (GF) animal models, gut microbiota manipulation with antibiotics and fecal microbial transplantation, have been performed to investigate the modulatory effect of the microbiota on gut–brain axis and its implications in neurodegeneration. Omics sciences, such as metagenomics, metatranscriptomics, metaproteomics, and metabolomics, represent the last frontier in elucidating host-microbiota cross-talks, providing invaluable contributions for unravelling this intriguing issue [10]. The integration of such systems biology-based approaches, supported by computational and bioinformatics analyses, help to shed light on the role of microbiota in the etiology and/or development of neurodegenerative disorders [11]. 

In this review, mainly taking into account peer-reviewed studies of the last five years, we aim to provide a general description of the state-of-the-art of the investigation methods used in the study of microbiota, with particular emphasis on meta-omics sciences such as metagenomics, metatranscriptomics, metaproteomics, and metabolomics, along with their complementary integration made feasible by the advances in bioinformatics tools. In addition, we focused on the latest achievements of these approaches in elucidating the influence of gut microbiota in the onset and/or progression of the most commonly studied forms of neurodegeneration, such as multiple sclerosis (MS), Alzheimer’s disease (AD), Parkinson’s disease (PD), and amyotrophic lateral sclerosis (ALS). 

## 2. Methodology

To achieve the stated aim, a detailed and in-depth search procedure was carried out. The literature analyzed in this review includes original studies available in qualified databases, such as Medline/Pubmed, Scopus, Web of Sciences, and Google Scholar. The literature searching and evaluation covers the last 20 years. However, it should be noted that the oldest references are related to the early advances in the omics sciences or definitions of diseases. The literature of the last 10 years was mainly considered throughout the manuscript. Instead, for the list of the latest achievements obtained from the application of meta-omics sciences (Table 1), only the last 5 years were taken into consideration.

A combination of key words and terms was used: microbiota/omics sciences; microbiota/ neurodegeneration; gut–brain axis/neurodegeneration. Each disease (MS, PD, AD, ALS) was combined with all the meta-omics sciences (metagenomis, metatranscriptomics, metaproteomics, metabolomics), respectively.

The references of all identified articles and recent review articles were cross-checked to ensure a valid and effective search.

## 3. The Microbiome Investigation in the “Meta-Omics Era”

The technological progress of the last decades has marked enormous changes in the methodology adopted for the investigation of the microbiota and its relationship with the host, moving from the traditional culture-based approach to the omics sciences. 

Conventional culture-based methods fail to identify all the microorganisms that make up the microbiota and are limited to analyze and elucidate up to only 10%–30% of the cultivated microbial community both in terms of composition and functions [43,44]. 

Based on a holistic perspective, omics and meta-omics sciences, including shotgun metagenomics, metatranscriptomics, metaproteomics, and metabolomics, use attractive and powerful tools to characterize the microbial consortia, investigate functions and dynamics, and quantify the biomolecules they produce. In this perspective, the resident microbial genomes (metagenome), transcripts (metatranscriptome), proteins (metaproteome), and metabolites (metabolome) are investigated in the human frame, providing a comprehensive overview of the complex network of interconnections that regulate the functional dynamics of each anatomical district [43,45].

These techniques provide detailed information on taxa strains of the microbial population, evaluate potential microbial functions and molecular networks, and quantify their protein and metabolic products. In addition, the application of these meta-omics approaches to clinical samples has identified microbial species, protein and metabolic pathways that could be associated with the development and treatment of human diseases. 

The main challenge of the omics-based microbiome studies relies on the strong computational effort required to deal with the impressive amount of data generated by the constantly improving next generation sequencing (NGS) and mass spectrometry (MS) technologies [46]. 

On the other hand, the integration of such omics sciences in the so-called multi-omics approach, provides more evidence of biological mechanisms and, ultimately, opens new perspectives for the development of novel therapeutic strategies and personalized medicine.

The technical aspects of omics sciences have been widely described elsewhere [10,47,48,49].

The following paragraphs will provide an overview of the elective technologies used in the study of the gut microbiota and its crosstalk with the brain, and how these disciplines have contributed to elucidate the link between the human microbiota and neurodegenerative diseases. 

### 3.1. Metagenomics

Metagenomics is a community-based powerful tool that studies the microbial genomes collected from the ecological niche (e.g., the gut) where the microorganisms coexist, in order to describe the phylogenetic, physical, and functional features of the microbiota, in a culture-independent manner. 

Current metagenomics techniques are based on the shotgun approach in order to provide a microbial community census starting from reads from DNA and alignment with reference genomes [43]. In parallel, phylogenetic composition of the microbial community has been mostly investigated by targeted sequencing of species-specific genes. 

Here, targeted sequencing of the 16S rRNA (prokaryotes) or 18S rRNA (eukaryotes) gene is one of the most attractive strategy for a reliable and cost-effective investigation of the overall microbial community diversity [50]. The obtained data are compared with curated taxa databases in order to cluster the analyzed reads into operational taxonomic units (OTUs) [51]. This “marker gene” approach, based mainly on PCR techniques, offers several advantages: it is time- and cost-efficient, and is very sensitive to the specific marker gene analyzed [49,50,52]. Similarly, it shows some limits. In fact, since the analysis concerns a single gene, important information such as the complexity of the entire microbial community in the sample and the potential functional characteristics related to taxonomic classes are lost [53].

Shotgun metagenomics, instead, by sequencing all the microbial genomes, allows for a more complete picture of the functional gut microbiota overcoming the marker-gene limitations [54].

The investigation of the whole metagenome is based on the construction of the metagenomics library following DNA extraction and cloning [55]. The metagenomics library is then subjected to sequence- and/or function driven-analysis. The first approach provides a catalogue of the identified genes and genetic elements (e.g., mobile genetic elements) to allow the taxonomical assessment of the microbiome; in addition the function-driven analysis also provides an in-depth prediction of the potential function of the microbial community as assessed through the investigation of the functions attributable to the identified genes [56]. 

Thus, in general, metagenomic sequencing not only provides accurate information on the microbial composition and family classification, but can also allow functional annotation, and gene de novo prediction [55,57]. Nevertheless, metagenomics results are limited to information on gene sequences without considering the functional effectors such as transcripts and protein products [43]. Furthermore, the metagenomics sequencing cannot discriminate between genes from actually active or even dead bacterial populations, hence cannot provide sufficient information about microbiota functions; rather draft the functional potential of a given microbial consortia [43]. Moreover, metagenomics data may not have a very high genome coverage losing information on less abundant microorganisms [55,58].

Metagenomics studies should be supported by the constant updating of curated reference databases and calculation sequencing algorithms. The choice of database is a fundamental step.

The Human Microbiome Project has provided a database of bacterial genome information useful to predict OUT functions [59]. Preferred curated databases to study the human gut microbiota include RefSeq [60] and MetaHit [61]. A wide number of bioinformatics tools are nowadays dealing with metagenomics sequences, enabling an integrative approach between omics data. Commonly used tools for metagenomics functional analysis are MEGAN (MEtaGenome ANalyzer) [62], IMG/M (Integrated Microbial Genomes/Metagenomes) [63], and MG-RAST (MetaGenome-Rapid Annotation using Subsystem Technology) [64], Kraken [65], MetaPhlAn [66], and TIPP [67]. In addition, MG-RAST and IMG/M are also used as data repositories and, along with NCBI (National Center for Biotechnological Information) and EBI (European Bioinformatics Institute) represent the biggest data repositories currently available. Although based on different algorithms and slightly different statistics, these enable the taxonomic and functional annotation of the metagenomic sequences, and allow the comparative evaluation of multiple datasets [68,69].

### 3.2. Metatranscriptomics

To overcome the metagenomics limits, metatranscriptomics is aimed at investigating the gene expression and activity of the whole microbial community [70,71]. 

Despite the wide choice of techniques, metatranscriptomics studies mainly focus on mRNA sequencing to assess which genes are expressed in the analyzed community [43]. The sequencing technology is the same as that adopted in metagenomics; however, key differences are represented by the selective removal of the interfering nucleic acids (e.g., DNA, t-RNA, rRNA) and the reverse transcription of m-RNA to cDNA prior to library production and its subsequent sequencing [71].

Some limitations are related to the difficulties of obtaining sufficient amounts of RNA to be analyzed and, mostly, separate mRNA from the other abundant RNA, such as rRNA. Lastly, metatranscriptomics classification is limited to not enough reference databases. 

As for metagenomics, bioinformatic data analysis is of fundamental importance for the comprehensive functional characterization of the RNA molecules. In this perspective, efforts are still required to improve and develop bioinformatics tools that integrate the metagenomics sequence with metatranscriptomics data [72]. 

Simple Annotation of Metatranscriptomes by Sequence Analysis (SAMSA) was the first open-source bioinformatics pipeline designed for metatranscriptomic data. It works with the metagenomics (MG) RAST server, a public resource for handling both metagenomic and metatranscriptomic data [73]. 

SAMSA’s latest implementation, SAMSA2, based on several tools such as DIAMOND for sequence alignment and local databases, manages end-to-end metatranscriptome analysis for stand-alone use on a computing cluster [74].

Along with MG-RAST, COMAN is a web-based tool to analyze metatrascriptomics data, although both do not support mapping to reference database. MetaTrans is another open-source pipeline as well as Anvi’o and IMP for the integrated metagenomics and metaproteomics analysis [75].

### 3.3. Metaproteomics

The important role of proteomics for the effective reliable identification of the metabolically active microorganisms has been widely recognized [76,77]. There are no doubts that protein identification is a key step to providing information on microbiota functions [76]. 

Metaproteomics (also known as Community Proteomics, Environmental Proteomics, or Community Proteogenomics) is the study of all protein samples recovered directly from environmental sources. 

Along with metabolomics, metaproteomics provides the most realistic picture of the key effectors that directly mediate the biochemical functions operated by the organisms at the specific moment of sampling [78,79].

The recent interest in the investigation of more complex samples, such as the gut microbiota, leads to the advent of high-performance MS platforms, which are now the dominant approaches for metaproteomics investigations. The actual MS technologies enable the investigation of different features of the metaproteome, such as differential proteins expression (e.g., time or treatment dependent), investigation of sub-proteomes (i.e., protein profile of the subcellular structures), post-translational modification (PTM) pattern, protein–protein interactions, and absolute protein quantitation [78,79]. Although several MS-based proteomics protocols have been optimized as widely reviewed [9,80,81], gut metaproteome is mainly investigated through a “bottom-up” approach, which allows to analyze entire proteomes starting by peptides obtained through a proteolytic digestion (typically by trypsin) from the extracted proteins. Peptides are usually separated first by liquid chromatography (LC) prior to their measurement at the mass spectrometer. Different methods of sample preparation and treatment have been developed to “resolve” the complex mixtures required for the MS analysis [76]. These are compatible with the advanced online separation technologies (such as nano Ultra Performance Liquid Chromatography, nUPLC) and provide a better separation of the complex mixture, thus improving protein detection rate [82]. 

Following computational assistance and using protein reference databases, MS data are processed to analyze the peptide sequences and, subsequently, provide protein identifications. 

A typical MS experiment generates hundreds of thousands of fragmentation spectra and an enormous amount of MS data that cannot be manually elaborated. Efficient bioinformatics software and tools are currently available to perform the computational operations required to translate the myriad of spectra into a meaningful output that is both concise and informative [78]. Commonly used search engines are OMSSA (Open Mass Spectrometry Search Algorithm) [83], X!Tandem [84], MASCOT [85], Andromeda [86], and SEQUEST [87]. Major tasks of these tools include quality filtering of the raw MS spectra, peptide-spectrum matching and scoring, protein database searching, data mining, and graphical representation of the obtained results [88].

Moreover, the bioinformatics data analysis and the availability of reference genome/metagenome sequences is undoubtedly of paramount importance in order to obtain a comprehensive picture of the metaproteome analyzed. 

Similarly to metatrascriptomics, the availability of enough reference databases can represent one of the technical limitations of this omics approach. In addition, robust, well-standardized protocols should be integrated in the experimental workflow especially for quantitative metaproteomics studies, which are still lacking [89].

### 3.4. Metabolomics

Metabolomics provides a snapshot of the metabolites array produced by the microbial community at the moment of sampling, enabling a comprehensive investigation of the microbial population and the interactions between the microbial ecosystem and its host [90]. Together with the knowledge about genetic products obtained from mRNA studies and the metaproteomic analysis, metabolomics draws the metabolomics profiles, elucidating the network of interactions between the host and its associated microbiota [43] as well as providing key information on the role of each microbial member in the onset and/or development of specific pathological conditions [91]. Identification of the metabolites catalogue enables a comprehensive elucidation of the ongoing physiological processes and the investigation of the cross-talks occurring between host–microbes and among microbial species [91,92].

The main steps in metabolome investigation include the pre-resolution of the complex metabolite mixture, following the sample lysis and/or purification of the extracted metabolites from samples of interest. Metabolites separation is generally accomplished through high performance liquid chromatography (HPLC) or gas chromatography (GC), enabling a wide range of metabolites to be analyzed through high sensitivity MS platforms. Similarly to metaproteomics, metabolites identification and their subsequent quantitation is computed on the basis of the mass spectral fingerprint and the MS fragmentation pattern.

Nevertheless, nuclear magnetic resonance (NMR) spectroscopy is also widely used in analyses that specifically target a reduced number of metabolites [93]. 

The metabolomics raw data requires complex processing steps to obtain small molecules and metabolite identification [94]. Several databases are available such as Golm Metabolome Database [95] and Metlin [96] for GC–MS and LC-MS data, respectively, while the Human Metabolome Database (HMDB) allows both LC-MS and GC–MS data identification analysis [97].

### 3.5. Multi-Omics Approach

As we previously described, each omics technology sheds light on an important aspect of the multifaceted microbial intestinal community, however, it is limited to a single perspective. Although each single approach has its own limits linked to technical issues, multi-omics integration can generate a comprehensive scenario that includes more evidence while explaining biological mechanisms and phenomena. 

In this context, insights integrated by combinations of different multi-omic sciences (metagenome, metatranscriptome, metaproteome, and metabolome) can provide a more detailed description of microbiota–host interactions, in order to reveal the bilateral flow of information that underlies different diseases, including neurodegeneration.

An important task in the field of multi-omics sciences concerns the annotation and integration of the identified molecules (DNA, RNA transcripts, proteins, and metabolites) into predicted classes, and clustering them into functional groups. For this purpose, data repositories commonly used are Kyoto Encyclopedia of Genes and Genomes (KEGG) [98,99], Gene Ontology (GO) [100], BioCarta. These provide information concerning the sub-cellular localization, biological process, and molecular function for each of the listed gene product. In addition, comprehensive repositories (e.g., KEGG), enable browsing of the literature available for each of the selected dataset entry, link it with known pathologies or pathogenetic mechanisms, and group the dataset entries into biochemical pathways, allowing for a thorough and deep study of the microbial community. Several software and web-based applications, such as Web MGA, Cytoscape, IPath, DAVID, and others are used to retrieve and integrate functional annotation from one or a plurality of sequence repositories, providing a more comprehensive functional annotation of the investigated microbiome [69].

## 4. The Gut–Brain Axis 

Gut microbiota is in close connection with the central nervous system (CNS) through the GBA, enabling a bidirectional communication from gut to brain and vice versa. Briefly, the GBA includes the following key components: the CNS, the autonomic nervous system (ANS), the enteric nervous system (ENS), the hypothalamic–pituitary–adrenal axis (HPA), the immune system (cytokine and chemokines) [101,102]. Complex connections develop both anatomically and biochemically, including direct and indirect pathways between the cognitive and emotional centers of the brain and peripheral intestinal functions. The ANS guides the afferent and efferent neural signals between the intestine and the brain. The HPA axis and the Vagus nerve with many spinal and vagal sensory neurons carry information from the intestinal end to the brain stem, which, in turn, includes the hypothalamus and the limbic system. Similarly, projections descending from the limbic system (HPA axis) influence the autonomic activity of the gut, especially under stress stimuli [101]. The intestinal microbiota plays a fundamental role in the development and maturation of both the human CNS and the ENS from the first postnatal weeks. For example, the interaction between the intestinal microbiome and the gastrointestinal mucosa membrane helps to refine and strengthen the developing immune system [103].

Biologically active neurochemical molecules of bacterial origin provide a mechanistic basis on how the microbiota may influence brain homeostasis and physiological processes [104].

Much research on the brain–gut microbiota axis is based on the use of GF animals. Many of these studies suggest that the intestinal microbiota produces relevant levels of neurotransmitters that are, in part, responsible for many aspects of brain health and disease [102,105]. For instance, several species of *Lactobacillus* and *Bifidobacterium* produce microbial metabolites, such as gamma-aminobutyric acid (GABA), which is the main inhibitory neurotransmitter in the brain. *Candida*, *Escherichia*, and *Enterococcus* produce the neurotransmitter serotonin (5-HT), while some species of *Bacillus* secrete dopamine. In particular, 5-HT has different roles. In the peripheral system, it is involved in the regulation of gastrointestinal secretion, motility (e.g., contraction and relaxation smooth muscle), and pain perception, while in the brain 5-HT is involved in regulating mood and cognition. The gut microbiota also plays an important role in the metabolism of tryptophan, precursor of 5-HT production, and in the stimulation of enterochromaffin cells, which are the main producers of 5-HT in the intestinal mucosa (about 95%) [102,106]. Bacteria fermenters produce short-chain fatty acid (SCFAs), such as butyric acid, propionic acid, and acetic acid, which are able to stimulate the sympathetic nervous system, the release of serotonin into the mucous membranes, and affect brain memory and learning processes [107]. In addition, bacterial products are able to stimulate enteroendocrine cells (EEC) to produce different neuropeptides, such as peptide YY, neuropeptide Y (NPY), and substance P, that can enter the bloodstream and affect the ENS [102].

A strong functional integrity and interplay of the GBA is required for the homeostasis of the nervous systems. Alterations of these connections in the GBA may influence the progress of neurodegeneration or even contribute to its onset [108,109].

### 4.1. Gut Microbiota and Neurodegeneration

Over recent years, accumulating evidence has suggested that the gut microbiota is involved in neurodegeneration. The alteration of the brain–gut microbiota homeostasis could worsen the etiology, the pathogenesis, and/or the progression of some disorders such as MS, PD, AD, ALS, and others [110]. 

Neurodegenerative diseases are multifaceted disorders in which a close interaction of genetic and environmental factors seem to initiate the pathological process. 

**Figure 1:**
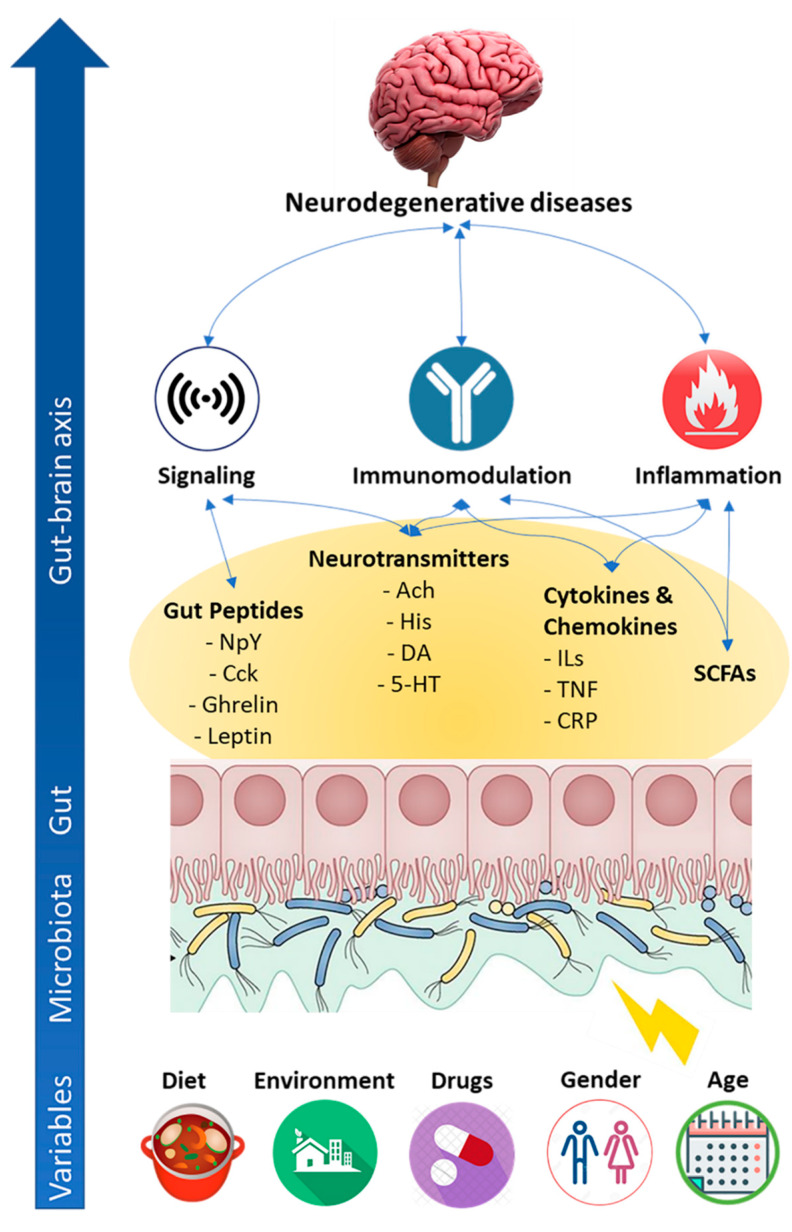
Major mechanisms employed by gut microbiota to impact neurodegenerative diseases. The figure depicts the most relevant endogenous (i.e., age, gender) and environmental (i.e., diet, environment, and drugs) variables affecting gut microbiota composition and functions. In turn, microbiota dysbiosis impacts neurodegenerative diseases through direct production of neuroactive molecules (e.g., short-chain fatty acid (SCFAs), neurotransmitters) and/or stimulation of neuroactive mediator production by the secretory epithelial cell (e.g., Citokynes, chemokine, gut peptides). Examples of neuroactive molecules are mentioned in the figure. Ach: Acetylcholine; His: Histidine; DA: Dopamine; 5-HT: Serotonin; NpY: Neuropeptide-Y; CcK: Cholecystokinin; ILs: Interleukins; TNF: Tumor Necrosis Factor; CRP: C-Reactive Protein.

Microbial metabolites and molecules trigger and/or amplify inflammatory brain processes, including perturbation of host immune homeostasis, alteration of blood–brain barrier and brain structure (Figure 1).

It is well known that oxidative stress (OS) is closely related to mitochondrial dysfunctions and is one of the main factors associated to neurodegenerations [111]. Interestingly, several lines of evidence showed that the microbiota can interact with host cells by merging with the mitochondrial activities [112,113,114]. Potential interactions between the microbiota–gut–brain axis and CNS oxidative stress could exist. Microbiota dysbiosis could increase the levels of reactive oxygen species (ROS) amplifying the OS scenario and neuronal inflammation. On the other hand, brain lesions, characteristic of various neuro-pathologies, can cause changes in gut microbiota composition and functions. The close correlation between OS–mitochondria–microbiota and neurodegenerative diseases sheds light on the importance of gut–brain axis connections [112]. 

Moreover, the reduced diversity of gut microbiota during aging, also influenced by dietary habits accumulated over the years, could have a role in the development of neurodegeneration. 

It is well known that composition of microbial community changes in diversity during aging [115]. The phyla *Bacteriodetes* and *Firmicutes* remain dominant, although their relative proportions may change significantly. An increase in pathogenic bacteria (pathobionts) usually occurs along with a concomitant decrease of beneficial bacteria (symbionts) [116].

Omics and multi-omics sciences support ongoing research studies to elucidate these intriguing connections and open new perspectives for therapeutic approaches for various neurodegenerative diseases.

The following sections describe the implications of an altered gut microbiota in some of the most relevant neurodegenerative disorders. An overview of some recent and relevant meta-omics studies has been proposed.

In Table 1, the most relevant meta-omics studies of the last 5 years are listed for each discussed disease. 

#### 4.1.1. Implications of Gut Microbiota in Multiple Sclerosis (MS) 

MS is an inflammatory disease characterized by immune-mediated axon demyelination leading to distinct signs, including autonomic and cognitive alterations as well as motor, sensory, and visual defects [117]. It is well known that the MS pathogenesis is strictly related to an impairment of the immune system, with significant contributions of other different factors including both genetic and environmental variables. Therefore, due to its function in the innate immune signaling, an involvement of the gut microbiota has also been proposed in MS [118,119]. 

Experimental evidence suggests that MS is also characterized by alterations of the intestinal permeability and bile acid metabolism which may consequently further contribute to complications in the immune regulation of the nervous system [119].

The role of intestinal dysbiosis in the onset or development of MS is currently not well understood. 

Experimental observations support the hypothesis of a cross-correlation between inflammatory demyelination of the CNS and modification of the microbiome. A few experimental evidences have been collected from analysis based on 16S rRNA sequencing on GF mice and experimental autoimmune encephalomyelitis (EAE) mice, a well-established mouse model of MS. [13]. In this cited study, by 16S rRNA sequencing, the authors show reductions in several bacterial components, such as *Lactobacillus*, that contribute to an impairment of immune system in fecal samples of EAE mice [13].

Recently, an integrated studied based on 16S rRNA sequencing and computational metabolomics has revealed that basis of the beneficial effect of the use of cannabidiol for the treatment of muscle spasticity of MS patients. The authors showed in EAE mice that cannabinoids directly prevent microbial dysbiosis, acting by the reduction of mucin degrading bacterial species, such as *Akkermansia muciniphila*, and consequently suppress neuroinflammatory processes [23].

Some few attractive studies have also been performed on human samples.

Through 16S rRNA biomarker sequencing, a characterization of gut microbiota composition has been carried out in fecal samples of pediatric MS compared to control children matched for age and sex. Perturbations in the gut microbial community composition have been shown in recent onset pediatric MS [21] and subsequent relapse risk [21].

Interestingly, two shotgun metagenomics based studies have been performed on cerebrospinal fluid of MS patients providing information about bacterial and viral composition also in such important biofluid [12,14].

In fact, previous research studies, based on marker-16sRNA gene, have highlighted a MS dysbiosis related to bacterial abundance such as *Faecalibacterium* [120] and *cClostridial* species [121] in fecal samples.

The use of antibiotics cocktails or probiotics, which clearly modify the composition of intestinal microflora commensals, improves immune responses as well as can generally attenuate the symptomatology of the disease [122].

These results provide a basis for future studies in which the controlled microbiota modulation could potentially contribute to the treatment of MS.

#### 4.1.2. Implications of Gut Microbiota in Parkinson’s Disease (PD)

PD is the second common worldwide neurodegenerative disease characterized by the death of dopaminergic neurons in the substantia nigra pars compacta (SNpc). Clinical symptoms generally appear when significant neuronal loss has already occurred. These include characteristic signs, especially related to the motor system, such as tremor, bradykinesia, rigidity, and postural instability. The recognition of these symptoms is the basis for a clinical diagnosis of advanced PD. Pharmacologic interventions are targeted at alleviating symptoms rather than preventing and/or resolving the disease [123]. Indeed, up to 30% of patients show non-motor symptoms, including olfactory and gastrointestinal impairments such as nausea, vomiting, and constipation [124,125,126]. The onset of constipation can also precede the motor symptoms and worsens with disease progression [127,128].

Growing experimental evidence focused on the microbiome demonstrating the potential role of gut microbiota on the disease state, as extensively reviewed in a recent paper by Sampson et al. [129].

As it is well known, Lewy bodies, characterized by toxic alpha-synuclein (αSyn) aggregates, are the main pathological hallmark of PD SNpc neurons [123]. It has been reported that αSyn deposition in neurons may begin in the neurons of the intestinal submucosa [130,131]. 

A large number of metagenomics studies have been focused on the identification of microbial taxa in PD gut microbiota, its taxonomic diversity and abundance. Many of these have been performed especially in fecal samples due to the non-invasiveness of the sample collection. Although some controversies occur in the results, it is well evident the idea of a clear correlation of alteration of gut microbiota in PD in comparison to a healthy status. In particular, 16S rRNA analysis showed higher prevalence of *Enterobacteriaceae* and reduced *Prevotellaceae* in fecal microbiota of PD patients compared to age-matched controls. Interestingly, the increased abundance of *Enterobacteriaceae* was correlated to the severity of postural instability and walking difficulties as well as a decrease of *Prevotellaceae*. Indeed, *Prevotella* produces SCFA, and thiamine and folate as by-products, that promote a healthy intestinal environment [25].

A shotgun metagenomic approach has been used by Bedarf et al. to investigate also the fecal microbiota of PD patients and healthy participants. The observed results revealed differences in the colonic microbiome composition and ẞ-glucuronate and tryptophan related metabolism at an unprecedented detail that was not achievable through other investigation techniques such as 16S rRNA gene sequencing [15].

Further metagenomics analysis has been shown that fecal microbiota of PD patients is featured by a decreased abundance of butyrate producing bacteria, known for their anti-inflammatory properties. Moreover, a parallel increase of pro-inflammatory *Proteobacteria* has been detected in PD mucosa [24].

An integrated metagenomics and metabolomics approach has highlighted that impaired concentrations of SCFAs, among the main metabolic products of gut-associated bacteria fermenters, may contribute to the gut microbiota-mediated alterations. Such alterations could worsen the ENS environment and, consequently, contribute to the gastrointestinal dysmotility of PD patients. Particularly, the reduction in SCFAs levels, along with a decrease of *Bacteroidetes* and *Prevotellaceae* and an increase of *Enterobacteriaceae* have been shown as the key players in PD fecal samples [27]. 

Studies performed on GF mouse models have been remarkably contributing to elucidating the molecular mechanisms underlying the microbiota–PD connection. As stated above, alterations of gut microbiota could be a trigger for αSyn aggregations [130,132]. 

Using αSyn overexpressing mice as a well-validated model for PD, it has been reported that gut microbiota promotes motor deficits, microglia activation and neuroinflammation, and ultimately αSyn accumulation [26]. Remarkably, colonization of αSyn-overexpressing mice with fecal microbes from PD patients show motor impairments in comparison to αSyn-mice treated with healthy feces. Microbiota composition of such mice highlighted taxa-related changes, specifically regarding the family *Enterobacteraceae*, and in particular *Proteus* spp. [26] which also is one of the main bacteria involved in the small intestinal bowel overgrowth (SIBO), a pathological condition of the small intestine. PD patients often show a SIBO-comorbidity related to worsening motor symptoms [133]. 

Following an innovative approach, Flores Saiffe Farías et al. performed an in-silico framework to associate metaproteins with the brain proteins expression through ontological labels. Out of the metaproteome-derived data, PD was found to be associated with selected bacterial taxa, and functional classes related with neuronal communication, DNA/RNA metabolism, and alterations in the Major Histocompatibility Complex-I [41]. 

In general, it must be taken into account that PD is a multifaceted disease due to a complex interaction among genetic and environmental factors. These studies underline a pivotal role of the microbiome dysbiosis in PD, and further investigations are desirable to determine how it could trigger and/or amplify the neuronal damage.

#### 4.1.3. Implications of Gut Microbiota in Alzheimer’s Disease (AD)

AD is a chronic neurodegenerative disease characterized by synapses loss and neuronal death leading to a progressive decline in cognitive function, loss of memory and, lately, to dementia. Deposition of amyloid beta peptide (Aβ) in plaques and accumulation of hyperphosphorylated tau in the so-called neurofibrillary tangle (NFT) are the main hallmarks of AD [134]. 

The mechanisms underlying these toxic depositions, which lead to an increased neuro-inflammation, are not fully understood, but it has been supposed an important role of the gut microbiota in this context [135].

Cattaneo et al. highlighted a close connection between brain amyloidosis and pro-inflammatory gut bacterial taxa. Following a deep metagenomics approach, patients with cognitive amyloidosis damage showed higher levels of pro-inflammatory cytokines, a rectal *Eubacterium* deficiency (known for the peculiar anti-inflammatory function) and an overabundance of *Escherichia/Shigella*, when compared to healthy patients. [19]. 

In GF Aβ precursor protein (APP) transgenic mouse model, a remarkable reduction of cerebral Aβ amyloid deposition has been shown when compared to control mice with intestinal microbiota. 

The absence of intestinal microbiota caused a significantly decreased cerebral Aβ amyloid pathology. Based on 16rRNA sequencing, the colonization experiments showed the importance of the nature of the donor (diseased transgenic versus wild-type model) for the promotion of AD. Thus, persistent gut microbial dysbiosis can regulate host innate immunity mechanisms that affects Aβ amyloidosis, and, consequently, microbiota modulation can induce positive effects on neuronal pathways, slowing down the progression of AD [33].

A recent study of Haran and colleagues has applied a metagenomics approach to comparatively evaluate the gut microbiome of AD elders versus the stool microbiome of non-AD elders. The study highlighted numerous microbial taxa and functional genes to be considered as predictors of AD dementia. Specifically, AD-microbiome is characterized by a lower proportion and prevalence of butyrate-producing bacteria along with a higher abundance of bacterial taxa commonly known to cause proinflammatory states [17].

#### 4.1.4. Implications of Gut Microbiota in Amyotrophic Lateral Sclerosis (ALS)

ALS is a neurodegenerative disease characterized by the death of lower and upper motor neurons in the spinal cord, brain stem, and motor cortex, leading to progressive paralysis and weakness. Although many factors are known to be implicated in the neuronal damage, such as microglia activated inflammation, neurotoxicity, redox unbalance, and a severe mitochondrial dysfunction, the deep and complex pathological mechanism affecting motor neuron is still not fully understood [111]. 

As widely reviewed by McCombe et al., theoretical reasons support the hypothesis of the involvement of gut microbiota in the pathogenesis of ALS. They include the important connections with the impaired metabolism, host immunity, and production of toxins that induce brain damage [54]. 

Most of the experimental evidence of gut dysbiosis comes from studies on mouse models for ALS, such as the SOD1^G93A^ mouse model. 

A dysfunction in the intestinal tract has been highlighted in transgenic SOD1^G93A^ mice, compared to wild-type mice, by Wu et al. Alterations in tight junction structure at the intestinal level and a reduced expression of the related protein (ZO-1), have been correlated to an increased intestinal permeability enabling the passage of toxins from intestinal lumen into blood circulation [136]. 

In ALS mouse altered microbiome composition and function have recently been highlighted, even before the onset of symptoms due to motor neuron dysfunction [20]. In particular, by shotgun metagenomics and 16S rRNA gene sequencing, it has been shown that the alteration of several strains, such as *Parabaceroides distasonis*, *Lactobacillus gasseri*, *Prevotella melaninogenica*, *Ruminococcus torques*, *Akkermansia muciniphila* (AM), and others, is related to disease condition. Interestingly, AM has been shown to decrease in a time dependent manner with the disease progression [20]. 

In general, a few studies have been performed on human ALS, and with conflicting results.

Brenner et al. have compared an ALS patient group, strictly selected without symptoms of dysphagia, gastrostomy, non-invasive ventilation, or low body mass index to an age- and gender-matched healthy control group. The metagenomics analysis of fecal microbiota has revealed that ALS patients do not show substantial alterations in gut microbiota composition, rather in the total number of microbial species and in the abundance of uncultured *Ruminococcaceae*. The authors concluded that ALS cannot undoubtedly be associated with a significant gut microbiota impairment [39].

Similarly, Rowin et al. showed a low diversity of intestinal microbial composition in fecal samples of ALS compared to controls, inferring that there is no direct and significant gut microbiota–disease correlation. Only a few ALS patients showed low *Ruminococcus* abundance, and low *Firmicutes/Bacteroidetes* (F/R) ratio, as an indicator of dysbiosis. In contrast, most patients showed inflammatory markers, such as fecal secretory IgA, eosinophilic protein X, and calprotectin. The authors therefore assumed that gut-mediated inflammation is likely to be involved in ALS onset or progression [38]. 

In a previous metagenomics study, a reduced *Firmicutes/Bacteroides* ratio has also been shown in ALS patients along with an increase of the abundance of genus *Dorea* and a decrease of the abundance of genus *Oscillibacter, Anaerostripes Lachnospiraceae* [37]. 

Conversely, Mazzini et al. highlighted an altered ALS gut microbiota by quantitative PCR analysis. Specifically, the authors showed a cluster distinction between bacterial profiles of ALS patients compared to controls, especially related to an increase of *Escherichia coli* and *Enterobacteria*, and a decrease of *Clostridium* and yeast [40].

Fecal microbiota transplantation (FMT) has emerged as a promising strategy to restore gut microbiota dysbiosis involved in complex pathologies including neurodegenerative diseases [137]. 

To this regard, a very recent paper has proposed a multicenter randomized double-blind clinical trial employing FMT as a therapeutic intervention for ALS patients at an early stage, opening new avenues for the treatment of neurodegenerative diseases by acting on the microbiota modulation [138]. 

## 5. Conclusions

It is nowadays well established that the gut microbiota is able to influence human health by acting on several physiological processes. In recent years, huge efforts have been made to shed light on the myriad of established cross-communications between the gut microbiota and distant organs, such as the brain. There is still an open debate about whether dysbiosis is a factor that determines neurodegeneration or rather an epiphenomenon resulting from it. 

The mutual correlation has undoubtedly been confirmed by many animal and human studies.

However, this field is still in its infancy and further complementary studies are needed to address the outstanding issues. 

In this context, the integrated approach based on the potential and experimental strength of the omics and meta-omics sciences currently opens new perspectives and provides powerful tools to support ongoing research and clinical studies. The valuable contribution meta-omics sciences have already made in the investigation of taxonomic characterization and functional dynamics of gut microbiota should be acknowledged. These sciences make it possible to obtain functional data and results not achievable with the other investigation methods available so far.

We are confident that the continuous technological progress and the development of innovative omics-based investigation methods will allow, in the near future, even more in-depth studies on a wide range of microbiota related disorders, including but not limited to neurodegenerative diseases.

## Figures and Tables

**Table 1:** Summary of relevant meta-omics studies correlating the gut microbiota to multiple sclerosis (MS), Parkinson’s disease (PD), Alzheimer’s disease (AD), and amyotrophic lateral sclerosis (ALS). The most recent studies are sorted depending on the method employed for the microbiota investigation.

Technique	Description	Pathology	Reference
Shotgun metagenomics	High-throughput method that provides information on the functional potential of the microbiota	MS	Perlejewski et al., 2016 [12] Colpitts et al., 2017 [13] Jovel et al., 2017 [14]
		PD	Bedarf et al., 2017 [15]
		AD	Sanguinetti et al., 2018 [16] Haran et al., 2019 [17] Park et al., 2017 [18] Cattaneo et al., 2017 [19]
		ALS	Blacher et al., 2019 [20]
Marker gene approach	PCR-based amplification of 16S/18S rRNA gene hypervariable regions	MS	Tremlett et al., 2016 [21] Tremlett et al., 2016 [22] Al-Ghezi et al., 2019 [23]
		PD	Keshavarzian et al., 2015 [24] Scheperjans et al., 2015 [25] Sampson et al., 2016 [26] Unger et al., 2016 [27] Hill- Burns et al., 2017 [28] Hopfner et al., 2017 [29] Heintz-Buschart et al., 2018 [30]
		AD	Minter et al., 2017 [31] Bonfili et al., 2017 [32] Harach et al., 2017 [33] Peng et al., 2018 [34] Xin et al., 2018 [35]
		ALS	Zhang et al.,2017 [36] Fang et al., 2016 [37] Rowin et al., 2017 [38] Brenner et al., 2018 [39] Mazzinì et al., 2018 [40]
Metatranscriptomics	High-throughput method that provides information on expression patterns of a given microbial community	ALS	Blacher et al., 2019 [20]
Metaproteomics	High-throughput method that provides information on the functional features of the microbial community proteins	PD	Flores Saiffe Farìas et al., 2018 [41]
Metabolomics	High-throughput method for the comprehensive study of the metabolite array resulting from the microbiota–host interactions	MS	Al-Ghezi et al., 2019 [23] Nourbakhsh B et al., 2018 [42]
		PD	Unger et al., 2016 [27]
		AD	Sanguinetti et al., 2018 [16] Xin et al., 2018 [35]
		ALS	Blacher et al., 2019 [20]
